# Root pull-out resistance and surface microstructural characteristics of adapted plants in the water-level fluctuation zone of the three parallel rivers area

**DOI:** 10.1371/journal.pone.0321597

**Published:** 2025-06-02

**Authors:** Zhong-liang Wang, Peng-biao Luo, Yuan-jun Yang, Ji-qi Duan, Qing-song Duan

**Affiliations:** 1 College of Resources and Environment, Yunnan Agricultural University, Kunming, Yunnan, China; 2 College of Water Resource, Yunnan Agricultural University, Kunming, Yunnan, China; VIT University, INDIA

## Abstract

Root pull-out resistance is an important index to measure the soil-fixing ability of roots. The study aims to investigate the root pull-out resistance and root surface microstructural characteristics of plants adapted to the Water-Level Fluctuation Zone (WLFZ) and provide a reference for the study of vegetation soil reinforcement capacity in the WLFZ of the Three Parallel Rivers area. The study subjects are the four-year-old *Arundo donax ‘Versicolor’*, *Cyperus involucratus*, and *Acorus calamus*. The study employs the single root pull-out resistance experiments to determine their resistance. Additionally, SEM and paraffin sectioning methods were utilized to measure the microstructure of the root surface and to explore the differences in microstructure and their impact on the friction between the root and soil. The findings revealed (1) The failure modes of the single root pull-out experiments included both pull-out and breakage, with 70.83%, 81.48%, and 57.69% of the roots being broken for *A. donax ‘Versicolor’*, *C. involucratus*, and *A. calamus*, respectively. (2)There were significant differences in the average maximum pull-out resistance and average frictional strength among the three plants (P < 0.05), with the average maximum pull-out resistance being *A. donax ‘Versicolor’* (27.88 N) > *C. involucratus* (20.53 N) > *A. calamus* (13.75 N), and the average frictional strength was *A. donax ‘Versicolor’* (43.48 Pa) > *C. involucratus* (31.77 Pa) > *A. calamus* (19.05 Pa). The root surface roughness also showed significant differences among the three plants (P < 0.05), with the surface roughness of *A. donax ‘Versicolor’* (20.13%) > *C. involucratus* (16.12%) > *A. calamus* (9.23%). (3) The root system of *A. donax ‘Versicolor’* was relatively rough, with dense depressions and protrusions. In contrast, the root system of *A. calamus* was relatively smooth with no significant depressions or protrusions, and *C. involucratus* was intermediate between the two. The results suggested that the maximum pull-out force of single roots for the three plants followed the order of *A. donax ‘Versicolor’* > *C. involucratus*> *A. calamus*. Moreover, the microstructure of the root surface had a significant impact on the maximum pull-out force of the roots, The rougher the root surface. The greater the single root drawing force.

## 1. Introduction

The Three Parallel Rivers area, located in the southwest part of the Qinghai-Tibet Plateau within the longitudinal valleys of the Hengduan Mountains, is an important ecological barrier in southwestern China. Within this region, the Nu River, the Lancang River, and the Jinsha River run parallel for 170 kilometers, with a significant elevation drop from the highest peak of Meili Snow Mountain at 6,740 meters to the Nu River valley at around 700 meters. Additionally, the area is characterized by active crustal movements and lies at the juncture of tectonic plates, with soft rock formations, extensive rock joints, and fragmented geological structures. These conditions facilitate the rapid weathering of soil parent material, causing frequent landslides, mudslides, and collapses, thereby classifying it as one of China’s high-incidence areas for soil erosion [1]. The Three Parallel Rivers area is rich in hydropower resources and has excellent development potential. So far, 23 large-scale hydropower stations have been planned and constructed in the area [2]. The operation of hydropower stations has created many large Water-Level Fluctuation Zone (WLFZ) along the banks of the three rivers. Indigenous vegetations are devastated due to the recurrent impacts of changing water levels, more extensive surface runoff caused by construction activities, and severe soil erosion near the reservoirs. Meanwhile, frequent local geological disasters threaten the safe operation of the hydropower stations.

The WLFZ refers to the geomorphological space between the lowest and highest water levels of rivers, lakes, and reservoirs, which is affected by seasonal water level fluctuations and human regulations. Its ecological condition is comparatively fragile [3–4]. Long-term alternation of dry and wet conditions leads to a decrease in the soil’s erosion resistance in the WLFZ, and vegetation restoration is an effective way to manage it. The interweaving of plant roots within the soil matrix exerts a “reinforcing and anchoring” influence, significantly bolstering the soil’s ability to resist erosion [5]. Previous studies have focused on selecting plant species well-adapted to the WLFZ, plant roots’ soil reinforcement capacity, and water submersion’s stress on plant growth [6–8]. However, most of these studies focused on the WLFZ of the Three Gorges Reservoir, with scant research available on the WLFZ in the Three Parallel Rivers area reservoirs. In contrast to the Three Gorges Reservoir area, the Three Parallel Rivers region boasts a higher altitude, more significant topographical relief, rugged terrain, profound river valleys, a vulnerable ecosystem, and is highly susceptible to soil erosion. Therefore, selecting plant species with a potent capacity for soil reinforcement is paramount for controlling soil and water loss in this area.

The anti-pulling ability of plant roots is considered the basic mechanism of soil-root interaction and an important index of root soil-fixing ability. When roots are pulled out, they may either be dislodged or fractured, with the specific form of damage contingent upon the tensile strength of the roots and the friction at the root-soil interface. Root tensile strength is affected by root diameter, root microstructure, and chemical composition. The friction between the root and soil interface is closely related to soil water content, soil bulk density, root diameter, root length, and root surface microstructure [9–12]. Studies have revealed that the friction force at the root-soil interface escalates with an increase in soil bulk density, root diameter, and root depth [13–14]. It initially rises and then declines with increased soil water content [15]. The microstructural characteristics of the root surface are essential in evaluating the friction characteristics of the root-soil interface. Studies have shown dense concave and convex structures on the root surface, which can significantly increase the friction force at the root-soil interface [16–18]. In addition, some scholars have used scanning electron microscopy images to show a chain model of the root system inside the soil, which is easy to attach to loose soil, which is attributed to root fiber and root surface roughness [19]. The above studies mainly focused on shrub plants (such as *Caragana korshinskii* and *Indigofera amblyantha*), and there was a lack of systematic analysis of the root surface microstructure of herbaceous plants and its relationship with root-soil interface friction characteristics. Herbaceous roots are mainly fibrous roots. Compared with shrubs, herbaceous roots have strong horizontal expansion ability, which can form dense shallow root networks quickly and improve soil erosion resistance through winding and reinforcement. The root morphology, epidermal tissue, and mechanical properties of herbaceous plants significantly differ from those of shrubs. However, the influence mechanism of root surface roughness and grid unit structure (such as concave and convex distribution) on root-soil friction is unclear.

In this study, three adapted herbaceous plants, *Arundo donax ‘versicolor’*, *Cyperus alternifolius*, and *Acorus calamus*, were used as the research objects in the WLFZ Huangdeng Hydropower Station on the Lancang River. The purpose of this study was to (1) determine the pull-out resistance of a single root of three plants, (2) determine the microstructure of the root surface of three plants, and (3) explore the influence of the root surface microstructure of three plants on the pull-out resistance of single root. We hypothesized that there are differences in the microstructure of the root surface of the three plants and that these differences have an important effect on the root pull-out resistance.

## 2. Materials and methods

### 2.1. Overview of the study area

**Fig 1:**
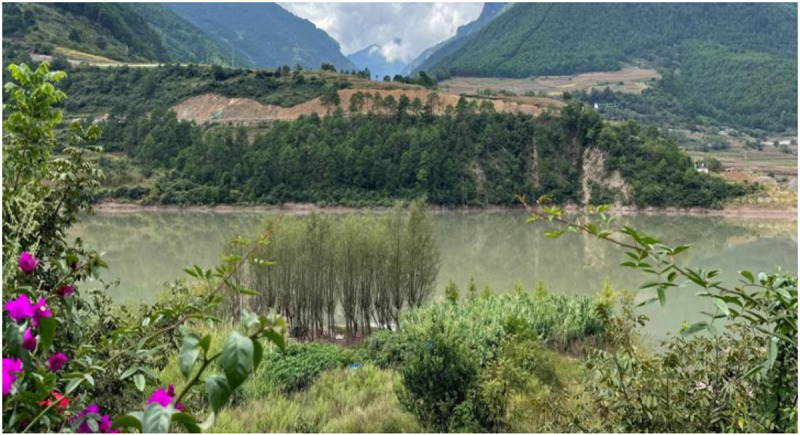
Experimental area in Weixi County.

The Huangdeng Hydropower Station is located in Lanping County, Yunnan Province, and was completed in June 2019 with an installed capacity of 1,900 MW. The power station reservoir runs through Lanping and Weixi counties. It has a standard storage level of 1,619 meters and a dead storage level of 1,586 m, forming a WLFZ with a height difference of 33 m. The Huang Deng Reservoir is situated in the Three Parallel Rivers’ core area and highly represents the region. The experimental area is located in Xiaozhuang Village (Fig 1), Wei Deng Township, Weixi County, on the left bank of the tail of the Huang Deng reservoir of the Lancang River, which is a gentle and open WLFZ (27°6′56″N, 99°10′29″E). The area is located at the end of the Biluo Snow Mountain range in the Hengduan Mountain range. It has a subtropical and temperate monsoon plateau mountain climate, with an average annual temperature of 14.9°C and an average annual precipitation of 938.6 mm. The soil type is sedimentary soil.

In May 2019, 16 types of herbaceous plants, including *Cynodon dactylon*, *Chrysopogon zizanioides*, *Phragmites australis*, *A. calamus*, *C. involucratus*, *Canna indica*, and *A. donax ‘Versicolor’*, were planted in the experimental area. After four years of continuous observation, it was found that *A. donax ‘Versicolor’*, *C. involucratus*, and *A. calamus* had the best growth. The coverage of *A. donax ‘versicolor’*, *C. alternifolius*, and *A. calamus* were 100%, 60%, and 75%, respectively.

### 2.2. Test materials

The soil samples used in the experiment are alluvial soil collected from the WLFZ area of the Huangdeng Hydropower Station on the Lancang River. Before collecting the soil samples, surface impurities were removed, and then the soil was taken from deeper layers. The collected soil samples were delivered to the Soil and Water Conservation Laboratory of Yunnan Agricultural University. The natural moisture content of the soil was measured to be 20.72%, and the bulk density was 1.36 g/cm³. The content of sand particles (0.02-2.0 mm), silt particles (0.002-0.02 mm), and clay particles (<0.002 mm) were 15.11%, 27.21%, and 57.23%, respectively.

**Fig 2:**
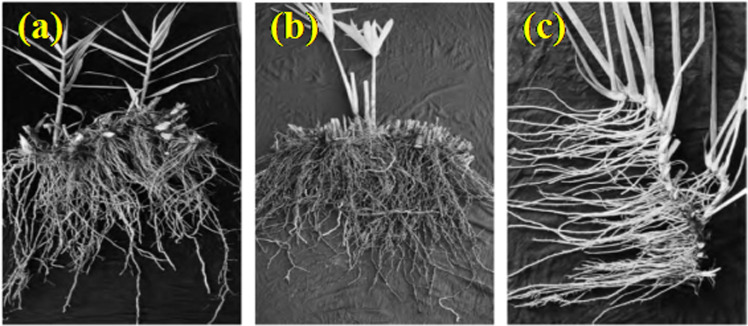
Root systems of three tested plant species. *(a)*, *Arundo donax ‘Versicolor’.* (b)*, Cyperus involucratus.* (c)*, Acorus calamus.*

The root systems of the three plants adapted to the WLFZ, *A. donax ‘Versicolor’*, *C. involucratus*, and *A. calamus*, were taken from the WLFZ of the Huang Deng Hydropower Station on the Lancang River in March 2023. They have been growing for four years and were sampled using the whole excavation method. After washing, they were returned to the laboratory and stored in a refrigerator at 4°C (Fig 2).

### 2.3. Single root tensile test

This experiment used a gauge length of 50 mm, and pull-out tests were conducted on 100 straight, undamaged single roots of each herbaceous plant. The diameter at both ends and the middle of the root system was measured using a digital vernier caliper, and the average diameter of the three positions was calculated as the diameter of the measured root system. The maximum tensile force of the single root was determined using the Sandu SN100 digital force tester (range 100 N, accuracy 0.05 N). Before the experiment, soft cloth tape was wrapped at both ends of the test machine’s clamps to increase the friction between the root system and the clamps. The distance between the upper and lower clamps was adjusted to 50 mm, and the root system was clamped. The test was started by hand at a uniform rotation of the test machine’s knob, with a stretching rate controlled between 10-15 mm/min. Data obtained when the root breaks at the middle one-third part of the test was considered successful during the test.

### 2.4. Single root pull-out test

**Fig 3:**
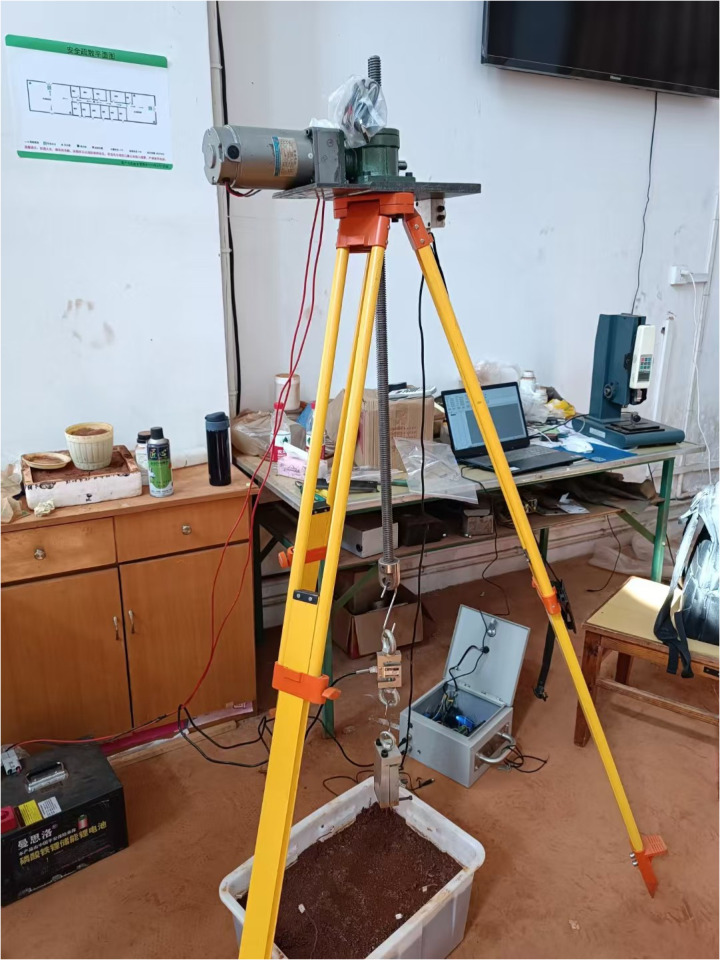
Schematic of the pull-out test apparatus.

Appropriate soil samples were placed into a testing box with dimensions of 300mm × 450mm × 250mm. The soil moisture content was controlled to be the same as that of the surrounding soil where the roots were sampled, which is 20.72%. The soil was compacted in four layers to an approximate bulk density of 1.36 g/cm³ (similar to the bulk density of the surrounding soil where the roots were sampled). Then, the single roots were vertically buried and compacted inside the testing box, with a free root end length of 50 mm and a burial depth of 150 mm. Subsequently, a self-designed pull-out apparatus was used to pull the roots at a 3 mm/min speed (Fig 3).

### 2.5. Observation of root surface microstructure

The root systems of herbaceous plants differ from those of shrubs in that they are prone to shrinkage after drying, which affects the observation of the root surface roughness. Therefore, in this experiment, the collected root segments were first fixed with FAA fixative solution (70% ethanol solution) for 24 hours, then dehydrated and embedded in paraffin wax, dried in a freeze dryer, and photographed and observed using an electron microscope.

After cleaning the collected root segments and cutting them into small sections, they were fixed with an FAA fixative solution. Following dehydration, embedding, sectioning, demolding, rehydration, safranin-fast green staining, and sealing, permanent sections were obtained. Subsequently, the NIKON Eclipse ci upright microscope was used for microscopic observation, and the NIKON digital sight DS-FI2 microscopic imaging software was used for photography. The images were processed using ImageJ software. A curve and a straight line were drawn along the concave-convex surface of the root, and the lengths of each line segment were measured. The root surface roughness was calculated using Equation (3), with five replicates for each plant.

### 2.6. Data processing and analysis

The calculation method for the tensile strength of a single root is as follows:


$$f=\frac{4F}{{\pi D}^{2}}$$ (1)

Where f is the tensile strength of the root (MPa), F is the maximum tensile force (N), and D is the root diameter (mm).

The frictional resistance per unit area of the root is represented by the frictional strength, satisfying [20]:


$$\tau =\frac{P}{\pi DL}$$ (2)

where P is the maximum pull-out force (N), D is the diameter (mm), L is the burial depth of the single root (mm), and π is the frictional strength (KPa).

The formula for calculating the surface roughness of the root is [16]:


$$T=\frac{L_{q}-L_{z}}{L_{z}}$$ (3)

In the formula, T is the root surface roughness, L_q_ is the total length of the curve on the root slice’s concave-convex surface (um), L_z_ is the total length of the straight line on the root slice surface (um).

Data were statistically analyzed using Excel 2021 and SPSS 20 and plotted using Origin 2018.

## 3. Results

### 3.1. Tensile properties of single roots of three plants

**Fig 4:**
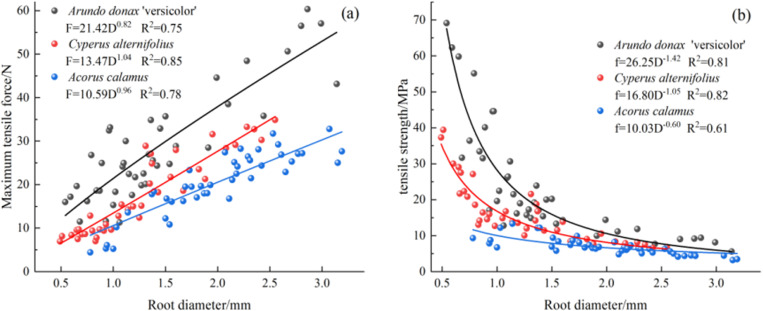
Variation curves of tensile force and tensile strength of single roots with root diameter in three plants. (a), the relationship between root diameter and maximum tension force. (b), the relationship between root diameter and maximum tension strength.

A total of 42, 39, and 43 roots of *A. donax ‘Versicolor’*, *C. involucratus*, and *A. calamus* were tested successfully, with diameter ranges of 0.54-3.14 mm, 0.49-2.55 mm, and 0.78-3.19 mm, respectively. The average diameters were in the order of *A. calamus* (1.99 mm) > *A. donax ‘Versicolor’* (1.43 mm) > *C. involucratus* (1.28 mm). As shown in Fig 4, the average tensile forces were in the order of *A. donax ‘Versicolor’* (28.39 N) > *A. calamus* (20.36 N) > *C. involucratus*(17.49 N). Moreover, the average tensile strengths were in the order of *A. donax ‘Versicolor’* (24.00 MPa) > *C. involucratus*(16.19 MPa) > *A. calamus* (7.01 MPa). The maximum tensile force increased with the increase in root diameter, while the tensile strength decreased with the increase in root diameter. The relationship between a single root’s tensile force, tensile strength, and root diameter could be fitted with a power function.

### 3.2. pull-out characteristics of single roots of three plants

During the three plants’ pull-out process, there were two modes of failure: pull-out and breakage, with breakage being the primary mode. As shown in Table 1, out of the successful tests of 24, 27, and 26 roots for *A. donax ‘Versicolor’*, *C. involucratus*, and *A. calamus*, respectively, 17, 22, and 15 roots were broken.

**Table 1:** Root pull-out Modes.

Plant species	Pull-out Failure/ Root	Breakage Failure/ Root	Test Number/ Root
*Arundo donax ‘Versicolor’*	7	17	24
*Cyperus involucratus*	5	22	27
*Acorus calamus*	11	15	26

**Fig 5:**
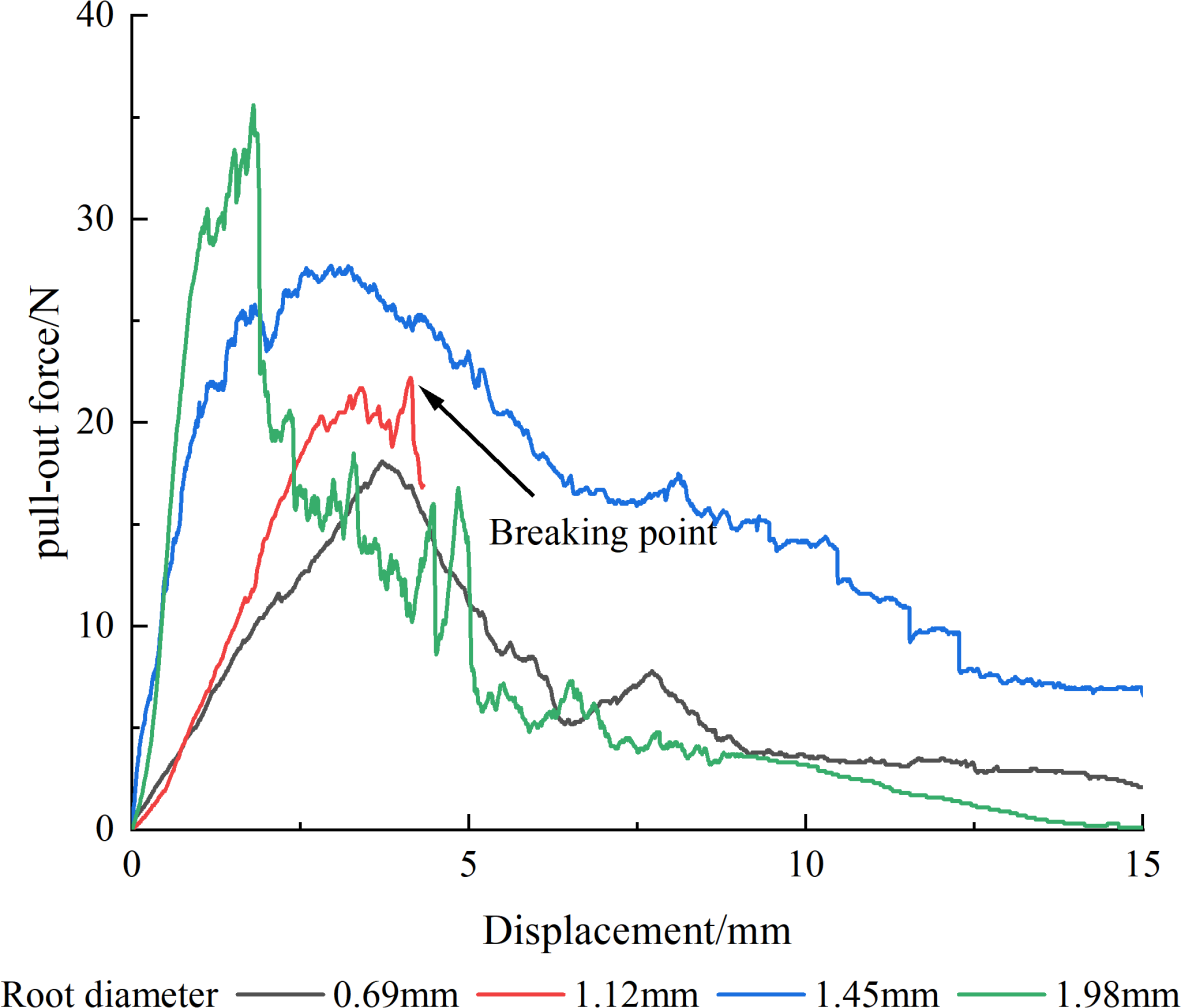
Pull-out force-displacement curves of single root pull-out tests for plants.

It was easily noticeable that the single root pull-out curve (Fig 5) had a clear main peak, and most curves have a single peak, with a few exceptions of multi-peak curves. During the pull-out failure, the pull-out force-displacement curve went through three stages: a sharp rise, a sharp drop, and a gentle decline. In the early stage of pull-out, as the sliding displacement increased, the friction between the root and soil also increased continuously until the connection structure of the root-soil interface and the overall stability were damaged, and the pull-out resistance reaches the highest value; as the load continues, the root-soil interface structure further damaged, and the soil particles rearranged, causing the friction area between the root and soil to shrink significantly, and the friction force begins to drop rapidly; as the root system continues to be pulled out, the root-soil interface structure was completely damaged, and the friction force between the root and soil slowly decreases until the root system was pulled out or there was only slight friction with the soil. During the breakage failure, the pull-out force-displacement curve goes through two stages: a sharp rise and a sharp drop. The trend of the sharp rise stage was consistent with the pull-out failure. When the friction force between the root and soil was greater than the tensile force of the root, the root was pulled off, so the peak value collected by this curve was not the maximum static friction force but the maximum tensile force of the single root itself.

**Fig 6:**
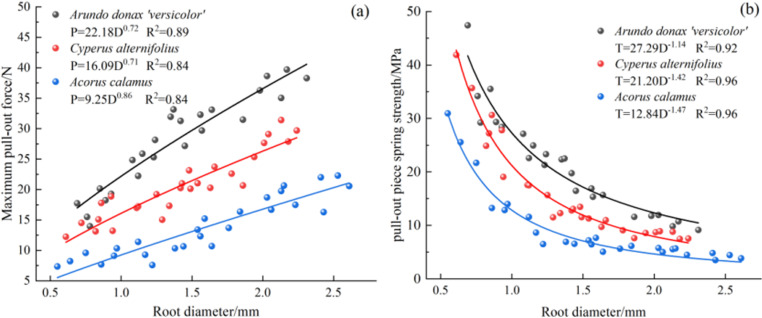
Curves showing the relationship between root diameter and maximum pull-out force and pull-out strength for three plants. (a), Root diameter-maximum pull-out force curves of three plants. (b), Root diameter-pull-out strength curves of three plants.

The diameter ranged of the successfully tested single roots for *A. donax ‘Versicolor’*, *C. involucratus*, and *A. calamus* were 0.69-2.31 mm, 0.61-2.24 mm, and 0.55-2.61 mm, respectively. The average root diameters were in the order of *A. calamus*(1.60 mm) > *C. involucratus*(1.43 mm) > *A. donax ‘Versicolor’* (1.40 mm). The pull-out force ranges for the three plants were 13.96-39.70 N, 12.23-31.41 N, and 7.35-22.29 N, respectively. The average maximum pull-out forces were in the order of *A. donax ‘Versicolor’* (27.88 N) > *C. involucratus*(20.53 N) > *A. calamus* (13.75 N). The pull-out strength ranges were 9.14-47.41 MPa, 7.54-41.87 MPa, and 3.51-30.95 MPa, respectively, with the average pull-out strengths being in the order of *A. donax ‘Versicolor’* (21.55 MPa) > *C. involucratus*(15.97 MPa) > *A. calamus* (9.22 MPa). The maximum pull-out force increased with the increase in root diameter, and the pull-out strength decreased with the increase in root diameter. The maximum pull-out force and pull-out strength could be well-fitted with a power function concerning root diameter (Fig 6).

### 3.3. Root-soil friction characteristics

**Fig 7:**
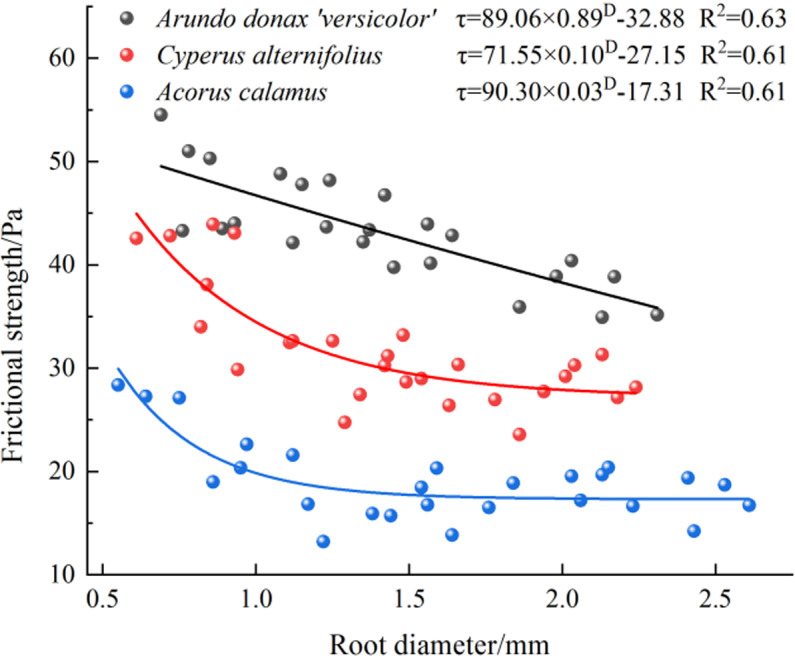
The fitted curve demonstrates the relationship between root diameter and frictional resistance intensity during vertical pull-out for three herbaceous plants.

According to Equation (2), the frictional resistance intensity of single roots was calculated and is shown in Fig 7. The frictional resistance intensity ranges for *A. donax ‘Versicolor’*, *C. involucratus*, and *A. calamus* were 34.92-54.52 Pa, 23.58-43.92 Pa, and 14.22-28.37 Pa, respectively. The average frictional resistance intensities were in the order of *A. donax ‘Versicolor’* (43.48 Pa) > *C. involucratus*(31.77 Pa) > *A. calamus* (19.05 Pa). The root diameter and friction strength were fitted by an exponential function, and the fitting effect was good.

### 3.4. Root surface microstructure

**Fig 8:**
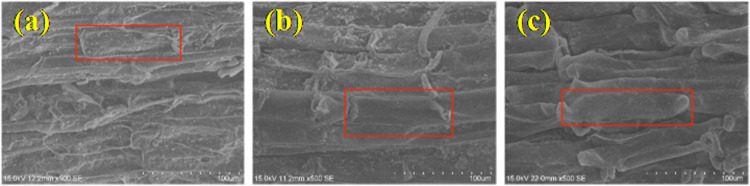
Root Surface Microstructure of the three plants (a)-(c). (a), *Arundo donax ‘Versicolor’*(× 500 times). (b), *Cyperus involucratus*(× 500 times). (c), *Acorus calamus*(× 500 times).

The roughness of the root surface determined the frictional characteristics between the root and soil. Many disordered combinations of relatively regular tetrahedral grid units could be observed on the root surface, perpendicular to the root axis, as shown in Fig 8(a–c). By measuring the grid size using ImageJ software, the major axis was 50-136 um, and the minor axis was 10-28 um. However, the roughness inside the grid units of the three plants was different. The grid units inside the *A. donax ‘Versicolor’* had noticeable depressions and protrusions, making the root surface uneven. In contrast, the grid units inside *C. involucratus* and *A. calamus* were smoother.

**Fig 9:**
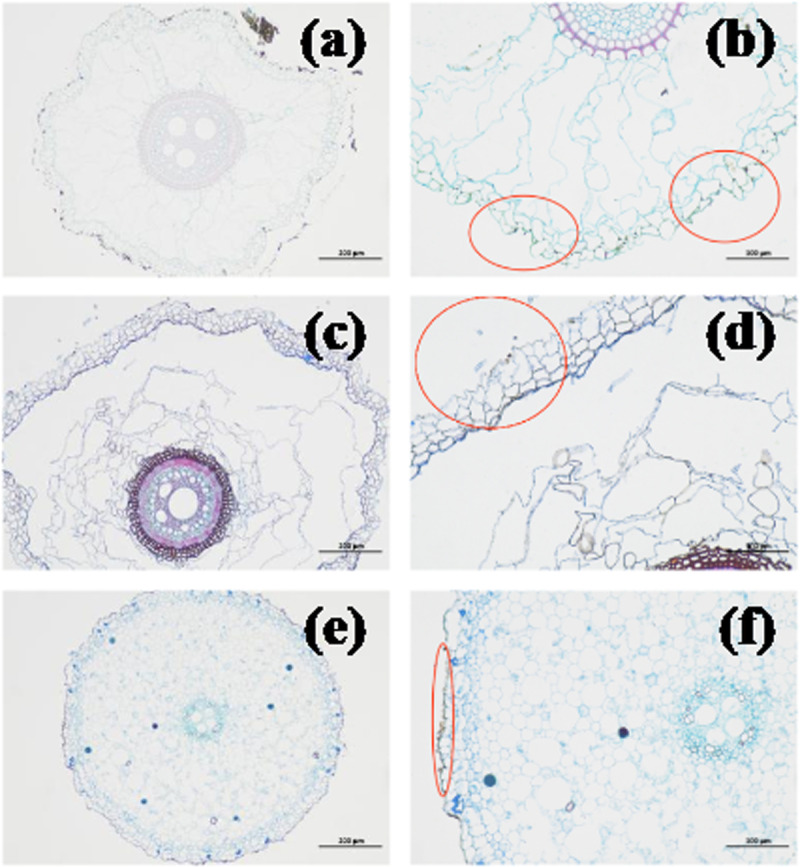
Root slices of the three plants (a)-(f). (a)-(b), *Arundo donax ‘Versicolor’.* (c)-(d), *Cyperus involucratus.* (e)-(f), *Acorus calamus*.

**Fig 10:**
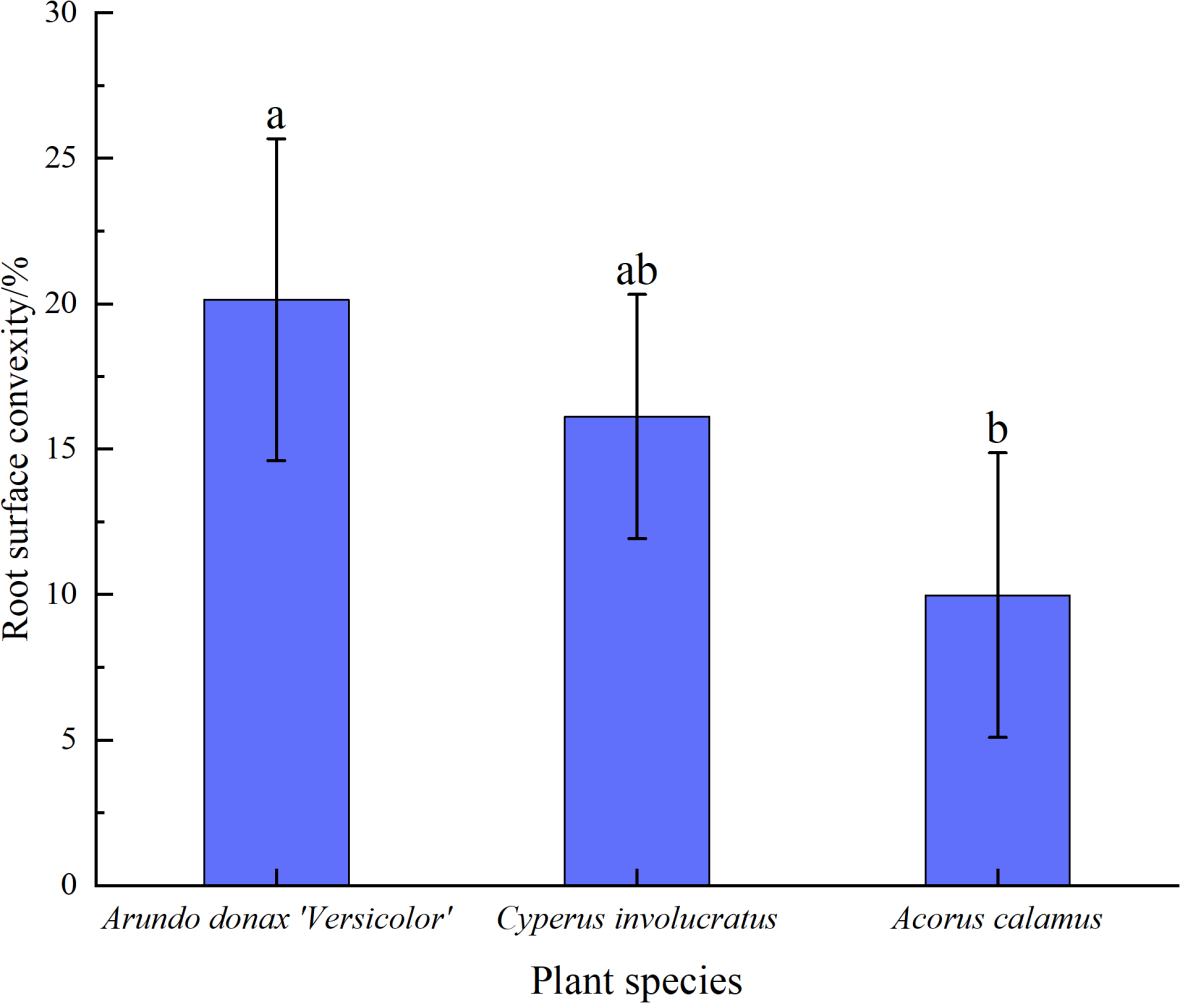
Surface roughness of the three plants’ roots. Notes: Different lowercase letters indicate significant differences among different plants (p < 0.05).

The roughness of the root surface was an important indicator of the frictional force between the root and the soil. The rougher the root surface, the greater the frictional and interlocking forces with the soil [16]. As shown in Fig 9, the root slices of the three plants revealed that the *A. donax ‘Versicolor’* had a rough root surface with numerous depressions and protrusions, *C. involucratus* had a relatively rough root surface with serrated structures, while *A. calamus* had a smoother root surface. The root surface roughness was measured, as shown in Fig 10, with the surface roughness being *A. donax ‘Versicolor’* (20.13%) > *C. involucratus*(16.12%) > *A. calamus* (9.23%), reaching a magnitude of significant difference.

Overall, there were significant differences in the root surfaces among the three plants. The *A. donax ‘Versicolor’* root surface was relatively rough with dense depressions and protrusions. When the contact surfaces slid against each other, fine soil particles could be firmly embedded in the dents on the root surface, increasing the friction between the root and soil. In contrast, the root surface of *A. calamus* was relatively smooth, with no apparent depressions or protrusions, making it difficult for soil particles to embed on the root surface, resulting in less friction between the root and soil. *C. involucratus* was intermediate between the two.

## 4. Discussion

### 4.1. Tensile and pull-out mechanical characteristics of three plant species

The maximum tensile force of the roots of the three plant species increased with root diameter following a power function. At the same time, the tensile strength decreased with root diameter, which also followed a power function. This finding is consistent with the research of other scholars [21–23]. Studies have shown [24] that the tensile strength of the root system is significantly positively correlated with the cellulose content in the root system. As the root system grows and the root diameter increases, the cellulose content decreases significantly, leading to a significant reduction in the tensile strength of the root system. It was found that the average maximum pull-out force of the three plants is in the order of *A. donax ‘Versicolor’* (28.39 N) > *A. calamus* (20.36 N) > *C. involucratus* (17.49 N). The *A. donax ‘Versicolor’*‘s average maximum pull-out force is 1.39 and 1.62 times that of *A. calamus* and *C. involucratus*, respectively. The average maximum pull-out force of *C. involucratus* ranks third, which is related to the smaller average diameter of the tested root system.

The maximum pull-out force of the three plants’ root systems increases with the increase of the root diameter following a power function. The pull-out strength decreases with the increase of the root diameter following a power function, which is consistent with the research on *Chrysopogon zizanioides* [21], *Medicago sativa* [25], and *Leonurus artemisia* [26]. As the root diameter increases, the friction area and friction force between the root and soil also increase, requiring a greater pull-out force to pull out the root system. There is a significant difference in the average maximum pull-out force of the three plants (P < 0.05), with the order being *A. donax ‘Versicolor’* (27.88 N) > *C. involucratus* (20.53 N) > *A. calamus* (13.75 N), and the maximum pull-out force of the *A. donax ‘Versicolor’* is 1.36 and 2.03 times that of *C. involucratus* and *A. calamus*, respectively. The root systems of the *A. donax ‘Versicolor’* and *C. involucratus* are more branched and dense, with developed root systems distributed in a reticular network, while *A. calamus* has a long, creeping rhizome, its fibrous roots growing uniformly downward from the rhizome, and the root system is thicker, with a single direction and sparse development. It can be inferred that the overall pull-out resistance of the *A. donax ‘Versicolor’* and *C. involucratus* is stronger than that of *A. calamus*. In addition, the root-soil composite shear strength of the *A. donax ‘Versicolor’* and *C. involucratus* is significantly better than that of A. Calamus [27], so the root systems of the *A. donax ‘Versicolor’* and *C. involucratus* have stronger soil reinforcement capabilities than *A. calamus*, and the *A. donax ‘Versicolor’* and *C. involucratus* can be preferentially planted under suitable conditions.

Single roots of the three plants exhibit two modes of failure during pull-out: pull-out and breakage, with breakage being predominant. The pull-out of *Betula platyphylla* single roots occurs in two modes, i.e., pull-out and breakage, under 13.85% moisture content and 1.58 g/cm³ dry density [28]. The roots of *Caragana korshinskii* exhibit three modes of pull-out: complete pull-out, fractured pull-out, and periderm slippage pull-out, under 15.1% moisture content and 1.65 g/cm³ density [12]. One pull-out failure mode occurred for *Senna bicapsularis* and *Indigofera amblyantha* under 17.15% moisture content and 1.43 g/cm³ density [29]. This variation is related to the characteristics of the plant root systems, soil types, and soil particle sizes. For the *A. donax ‘Versicolor’*, *C. involucratus*, and *A. calamus*, 70.83%, 81.48%, and 57.69% of the roots were broken, respectively. *A. donax ‘Versicolor’* and *C. involucratus* are more likely to break than *A. calamus*. On the one hand, the frictional resistance of the root systems of the *A. donax ‘Versicolor’* and *C. involucratus* is greater than that of *A. calamus*. On the other hand, this is related to the thin-walled cells of the root cortex. The thin-walled cells of the root cortex of *A. calamus* are more developed, while those of the *A. donax ‘Versicolor’* and *C. involucratus* are less so. During the pull-out process, the outer and inner cortexes are prone to separation, making the roots more susceptible to breakage.

**Fig 11:**
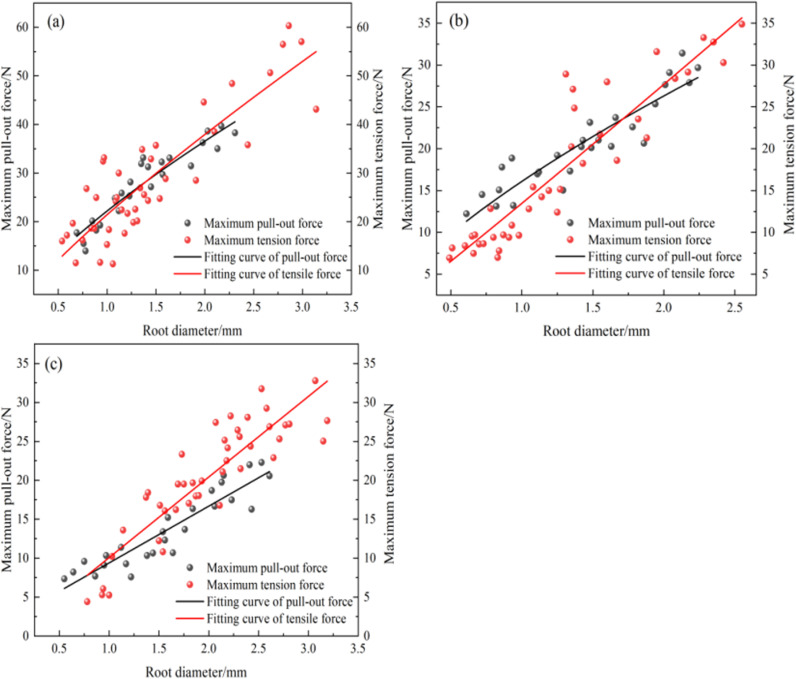
Comparison of single root tensile force and pull-out force. *(a)*, *Arundo donax* ‘Versicolor’. *(b)*, *Cyperus involucratus.* (c)*, Acorus calamus.*

Comparing the maximum pull-out force of the plant single roots with the maximum tensile force (Fig 11), it can be observed that there is an intersection (critical value) between the fitting curves of the maximum pull-out force and the maximum tensile force. When the root diameter is smaller than this intersection, the maximum tensile force of the single root is smaller than the maximum pull-out force, resulting in breakage failure. Meanwhile, when the root diameter is greater than this intersection, the maximum tensile force of the single root is greater than the maximum pull-out force, leading to pull-out failure [30]. The theoretical critical root diameters for the *A. donax ‘Versicolor’*, *C. involucratus*, and *A. calamus* are 1.41 mm, 1.71 mm, and 0.78 mm, respectively, with theoretical pull-out forces of 28.42 N, 23.68 N, and 7.98 N. However, the actual maximum root diameters at breakage are 1.98 mm, 2.13 mm, and 2.06 mm, with corresponding pull-out forces of 41.27 N, 36.41 N, and 21.68 N. It is evident that the actual diameters at breakage are greater than the theoretical values, and the actual pull-out forces at breakage are also greater than the theoretical forces. Studies have shown [31] that the ends of the roots are more likely to break during the pull-out process instead of in the middle. In this study, only the data where the breakage occurred near the middle 1/3 was considered valid during the tensile test of the single roots. The data was discarded when the breakage was far from the middle part, leading to an overestimation of the tensile test data and, thus, an underestimation of the theoretical critical values.

### 4.2. Microstructure of the root surface and frictional properties at the root-soil interface

The relationship between the root diameter and the frictional resistance intensity between the roots of the three plants is negatively correlated, which is consistent with the study on *Chrysopogon zizanioides* [32]. There is a significant difference in the average frictional resistance intensity among the three plants (P < 0.05), with the values being *A. donax ‘Versicolor’* (43.48 Pa) > *C. involucratus* (31.77 Pa) > *A. calamus* (19.05 Pa). The average frictional resistance intensity of the *A. donax ‘Versicolor’* is 1.37 and 2.28 times that of *C. involucratus* and *A. calamus*, respectively, which is related to the roughness of the root surface of the three plants. Through SEM scanning and sectioning, both transverse and longitudinal observations indicate that the *A. donax ‘Versicolor’* root surface is relatively rough with dense depressions and protrusions. Conversely, the root surface of *A. calamus* is relatively smooth without prominent depressions or protrusions, and *C. involucratus* is intermediate between the two. Quantitative analysis of the surface roughness of the three shows significant differences, with the *A. donax ‘Versicolor’* (20.13%) > *C. involucratus* (16.12%) > *A. calamus* (9.23%). Therefore, the significant difference in the average frictional resistance intensity among the three plants is due to the significant difference in the roughness of their root surfaces.

The difference in the roughness of the root surfaces of the three plants is mainly reflected in the internal structure of the grid units. The root surfaces have many disordered combinations of relatively regular tetrahedral grid units. The *A. donax ‘Versicolor’* grid units have apparent depressions and protrusions, which are not present in *C. involucratus* and *A. calamus*, resulting in a greater roughness of the root surface than that of *C. involucratus* and *A. calamus*. The root surface of *Caragana korshinskii* also has some tetrahedral, pentagonal, and hexagonal grid units [17]. However, the internal structure of the grid units is relatively smooth, which is different from that of the *A. donax ‘Versicolor’.*

## 5. Conclusions

The findings revealed two modes of pull-out failure: (1) pull-out and breakage, with 70.83%, 81.48%, and 57.69% of the roots being broken for the *A. donax ‘Versicolor’*, *C. involucratus*, and *A. calamus*, respectively. (2)There were significant differences in the average maximum pull-out force and average frictional resistance intensity among the three plants (P < 0.05). The average maximum pull-out forces were in the order of *A. donax ‘Versicolor’* (27.88 N) > *C. involucratus* (20.53 N) > *A. calamus* (13.75 N), and the average frictional resistance intensities were in the order of *A. donax ‘Versicolor’* (43.48 Pa) > *C. involucratus* (31.77 Pa) > *A. calamus* (19.05 Pa). (3)There were significant differences in the root surface roughness among the three plants (P < 0.05). The surface roughness was in the order of *A. donax ‘Versicolor’* (20.13%) > *C. involucratus* (16.12%) > *A. calamus* (9.23%). The *A. donax ‘Versicolor’* root system was relatively rough, with dense depressions and protrusions. Conversely, the root system of *A. calamus* was relatively smooth without noticeable depressions or protrusions, and *C. involucratus* was intermediate between the two. The effect of dry-wet alternation in the WLFZ is noticeable. In the future, the research on the effect of dry-wet alternation on the root-soil interface should be strengthened. Integrating multi-disciplinary, in-depth mechanism analysis provides a scientific basis for constructing ecological engineering in the WLFZ.

## Supporting information

S1 File Minimal data set. (XLSX)
